# Recent advances and role of melatonin in post-harvest quality preservation of shiitake (*Lentinula edodes*)

**DOI:** 10.3389/fnut.2024.1348235

**Published:** 2024-03-20

**Authors:** Hafiz Umair Asdullah, Feng Chen, Muhammad A. Hassan, Asad Abbas, Shoukat Sajad, Muhammad Rafiq, Muhammad Adnan Raza, Arslan Tahir, Dongliang Wang, Yougen Chen

**Affiliations:** ^1^School of Horticulture, Anhui Agricultural University, Hefei, China; ^2^Wandong Comprehensive Experimental Station, New Rural Development Institute, Anhui Agricultural University, Minguang, China; ^3^Anhui Academy of Agricultural Sciences, Hefei, China; ^4^School of Science, Western Sydney University Hawkesbury, Sydney, NSW, Australia; ^5^Lushan Botanical Garden of Chinese Academy of Science, Jiujiang, China; ^6^University College of Agriculture, University of Sargodha, Sargodha, Pakistan

**Keywords:** *Lentinula edodes* (shiitake), shelf life, post-harvest preservation, melatonin, quality control bioactive compounds

## Abstract

Shiitake mushrooms are renowned for their popularity and robust nutritional value, are susceptible to spoilage due to their inherent biodegradability. Nevertheless, because of their lack of protection, these mushrooms have a short shelf life. Throughout the post-harvest phase, mushrooms experience a persistent decline in quality. This is evidenced by changes such as discoloration, reduced moisture content, texture changes, an increase in microbial count, and the depletion of nutrients and flavor. Ensuring postharvest quality preservation and prolonging mushroom shelf life necessitates the utilization of post-harvest preservation techniques, including physical, chemical, and thermal processes. This review provides a comprehensive overview of the deterioration processes affecting mushroom quality, covering elements such as moisture loss, discoloration, texture alterations, increased microbial count, and the depletion of nutrients and flavor. It also explores the key factors influencing these processes, such as temperature, relative humidity, water activity, and respiration rate. Furthermore, the review delves into recent progress in preserving mushrooms through techniques such as drying, cooling, packaging, irradiation, washing, and coating.

## 1 Introduction

Mushrooms have a long-standing reputation as a highly nutritious food source and traditional remedy. There are approximately 14,000 mushroom species, with an estimated 3,000 considered suitable for consumption, and about 270 of these possess the potential to offer therapeutic benefits and enhance human health (1). Additionally, mushrooms serve as an excellent source of nutraceuticals, including ascorbic acid, carotenoids, tocopherols, phenolic compounds, and unsaturated fatty acids (2). *Lentinula edodes*, commonly known as shiitake, ranks among the most widely cultivated edible mushrooms globally (3). Its popularity has surged in both the international food industry and medical research sectors due to its abundant nutritious and therapeutic properties. Renowned for their unique taste, nutritional profile, and health benefits, shiitake mushrooms are prominently featured in various global cuisines (4). Shiitake mushrooms are rich in polysaccharides (primarily glucans like lentinan), dietary fibers, proteins, vitamins, essential amino acids, sterols for flavoring, and other nutrients. Furthermore, shiitake mushrooms are recognized as one of the top sources of ergosterol, a precursor to vitamin D₂ in the human body (5). It contains bioactive compounds with antibacterial and anti-carcinogenic properties, aiding in the prevention of liver cirrhosis and reduction of blood cholesterol levels (6).

*Lentinula edodes* lacks a protective cuticle layer on its skin, rendering it susceptible to both physical and microbial damage. Studies have indicated that the shelf-life of shiitake mushrooms varies depending on storage conditions: approximately 1 to 3 days at room temperature (20 to 25°C), 5 to 7 days at 0 to 2°C, and around 8 days under refrigeration (7). Mushrooms’ short shelf life hampers their economic worth. Throughout the post-harvest period, mushrooms undergo a sequence of quality deteriorations, including discoloration, moisture reduction, taste degradation, texture modifications, and nutrient depletion (8). Fresh mushrooms typically contain substantial moisture content ranging from 85 to 95%. This elevated moisture level creates an optimal environment for microbial proliferation, necessitating mushroom storage at lower temperatures to mitigate contamination risks. Throughout the post-harvest phase, mushrooms experience a gradual reduction in moisture content, leading to ongoing weight loss (9).

Various factors affect the quality of mushrooms after harvest, which can be categorized into internal factors concerning the mushroom itself, such as water activity, respiration rate, and microbial activity, and external factors concerning storage conditions, such as temperature and humidity. Numerous preservation techniques have the potential to minimize the deterioration of post-harvest quality and extend mushroom shelf life. Traditional methods like drying and cooling, categorized as thermal processes, are commonly used to slow down mushroom quality degradation by regulating storage temperature and water activity (10). Modified atmosphere packaging (MAP) is an alternative method that preserves fresh mushrooms’ postharvest quality (11). Furthermore, various chemical and physical processes, including irradiation (12), washing with antimicrobial agents, pulsed electric field treatment (13, 14), coating (15), ozone treatment (16), and electrolyzed water treatments (17), have been shown to effectively deactivate microbial activities and impact physical properties such as texture, color, and weight loss.

The current review emphasizes on highlighting recent progress being made in postharvest technology which includes the application of chemical treatment, modified atmosphere packaging (MAP), thermal dehydration methods and the application of essential oils and botanical extracts for various purposes along with their impacts on the physical, biochemical, nutritional, and sensory attributes of shiitake mushrooms. This study begins by elucidating the factors contributing to the degradation of different quality aspects of shiitake mushrooms. It explores the influence of various processing techniques on fresh and dried shiitake mushrooms. Additionally, it assesses the existing scientific literature and proposes concurrent methods to enhance the quality of both freshly harvested and dried shiitake mushrooms.

## 2 Functional mushroom

Functional mushrooms, such as Shiitake (*Lentinula edodes*), Reishi (*Ganoderma lucidum*), Lion’s mane (*Hericium erinaceus*), Chaga (*Inonotus obliquus*), Cordyceps (*Cordyceps sinensis*), and Turkey tail (*Trametes versicolor*), have garnered significant attention for their potential health benefits (18–20). These mushrooms contain a variety of bioactive compounds, including polysaccharides, terpenoids, and phenolic compounds. These compounds have been studied for their immunomodulating, anti-inflammatory, neuroprotective, and anti-cancer properties. For instance, Shiitake mushrooms are known for their immune-boosting effects due to compounds like lentinan and eritadenine, while reishi mushrooms have been traditionally used for longevity and exhibit antioxidant and immune-modulating properties. Lion’s mane mushrooms show promise in supporting cognitive health, particularly in conditions like Alzheimer’s disease. This is attributed to compounds like hericenones and erinacines. Cordyceps mushrooms are valued for their potential to enhance energy levels and athletic performance. Turkey tail mushrooms have been studied for their immune-enhancing and anti-cancer effects. Despite their promising attributes, further research is needed to fully elucidate the mechanisms of action and therapeutic potential of these functional mushrooms. Chaga mushrooms contain a variety of bioactive components such as polysaccharides, antioxidants, and melanin, which offer numerous health advantages. Its ability to modulate the immune system helps maintain its balance, while its anti-inflammatory characteristics reduce inflammation. With its abundance of antioxidants chaga effectively combats free radicals, protecting the cells from oxidative damage (Table 1).

**Table 1:** Contrasting shiitake mushrooms with different mushroom types regarding their medicinal qualities and biochemical composition.

Medicinal properties and biochemical composition	*Lentinula edodes* (Shiitake)	*Ganoderma lucidum* (Reishi)	*Trametes versicolor* (Turkey Tail)	*Ophiocordyceps sinensis* (Cordyceps)	*Inonotus obliquus* (Chaga)	*Hericium erinaceus* (Lion’s Mane)
Bioactive compounds	Beta-glucans such as lentinan, Polysaccharides, Glycoproteins, Phenols, Terpenoids, Nucleotides and Steroids	Acrylamides, Lectins, or glycoproteins phenolics, Triterpenoids, Nucleotides, Fatty acids, Vitamins and Minerals	Fatty acids (oleic, linoleic, linolenic, stearic,), Vitamin B, Polyphenols (phenolic acids: p-hydroxy benzoic, protocatechuic, vanillic, and homogentisic), Polysaccharopeptide and polysaccharide, (1,3) (1,6)- D-glucans	Cordymin (peptide), Adenosine, Trehalose, Cordycepic acid (D-mannitol), Saponins, Polysaccharide,Beta glucans, Ergoster-tocopherol Hydroxybenzoic acid, Polyunsaturated fatty acids and Cordycepin (purine alkaloid)	Fatty acids, Hydroxy acids, Triterpenoids (lanosterol), Polysaccharides, Steroids (ergosterol and ergosterol peroxide), coumarins, quinones, and styrylpyrones, Polyphenols (flavonoids and phenolic acids)	Volatile compounds (hexadecenoic acid, linoleic acid, phenylacetaldehyde, benzaldehyde), Lactone, Polysaccharides, Beta-glucans, Glycoprotein, Erinacins, Fatty acids, Sterols, Hericerins
Beneficial effects on health	Boosting immune function, Antimicrobial, Antioxidant, Antitumor, Antiaging, Hypocholesterolemic effect, Lowering of blood pressure	Hepatoprotective neurotonic, Immunomodelling nephrotonic, Hypotensive effect, Cardiotonic, Antimicrobial, Anticancer, Antiviral (including HIV), Anti-asthmatic, Anti-inflammatory effects	Prevent obesity, Antidiabetic, Antimicrobial, Antitumor, Antioxidant activity, Immunoregulatory	Hypocholesterolemic effect, Cardiovascular protection, Antioxidant, Anticonvulsant activity, Hypoglycemic effect, Antitumor, Anti-inflammatory	Cardioprotective effects, Antiglication effect, Antitumor activity, Anti-inflammatory hypoglycemic effect, Antilipidemic effect, Antioxidant, Anti-aging	Hypocholesterolemic effects, Neurotonic, Anti-asmatic, Hypoglycemic effects, Anticancer, Anti-aging, Antioxidant
β-glucan content (g/100 g on dry basis)	20.0 to 24.9	4.3 to 22.7	59.58	3.78	7.85	35.3
Protein	17.4 to 27.09	13.5 to 23.6	11.07	21.9 to 23.3	2.4	22.4
Lipids	1.26 to 2.95	3 to 5.8	1.35	5.5 to 8.2	1.7	3.5
Carbohydrates	37.9 to 65.9	43 to 81.9	37.7	23.7 to 50	11	57.8
Dietary fibers	46.13 to 48.76	15.12	30	7.9	66. 94	3.4 to 7.9
References	(13, 21, 22)	(23–26)	(27–29)	(30–32)	(25, 33, 34)	(26, 30, 35, 36)

## 3 Mushroom quality degradation

**Figure 1:**
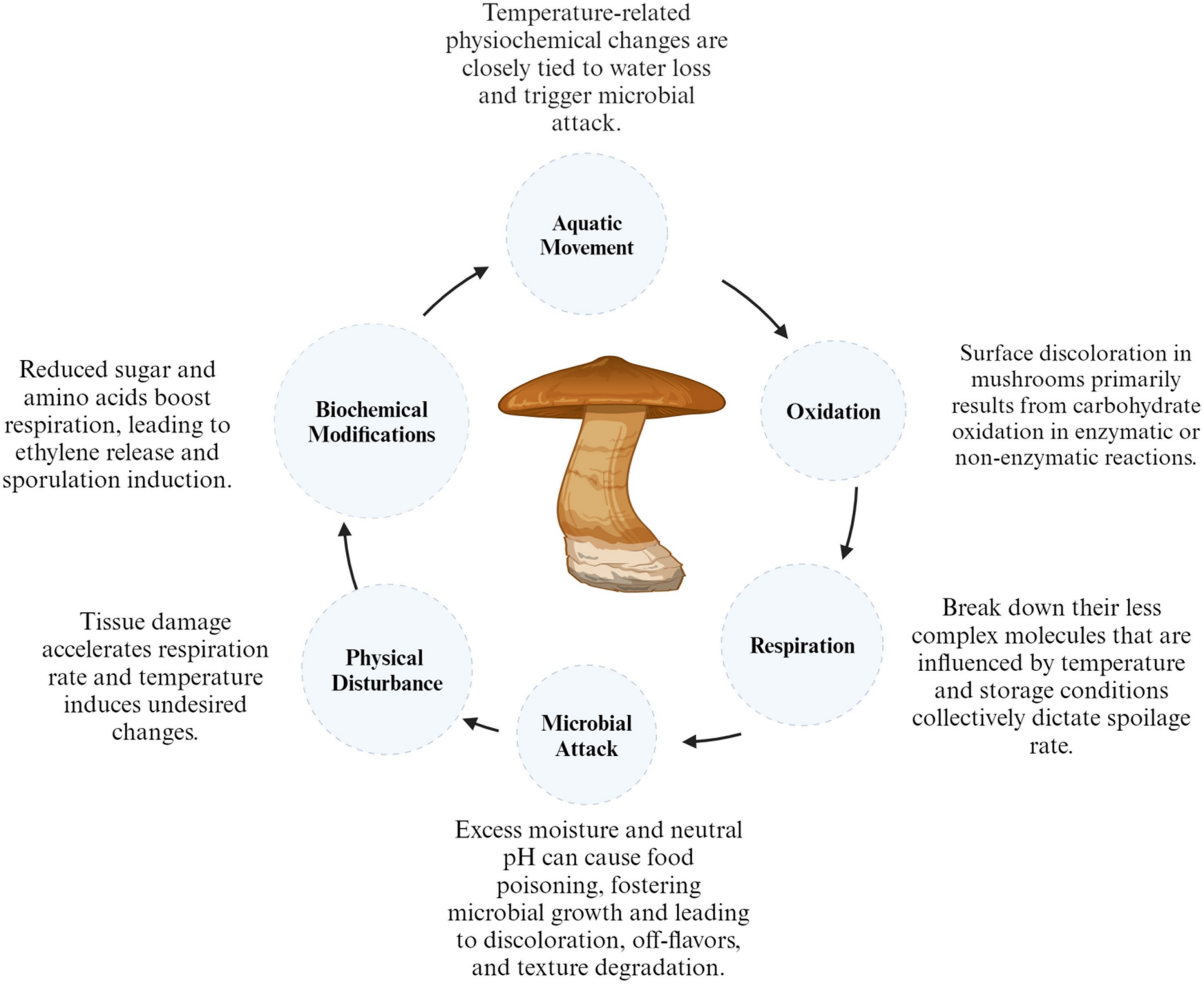
Factors effecting shiitake mushroom post-harvest quality.

Quality degradation in mushrooms occurs during the post-harvest period. The factors that commonly influence their quality include color changes, loss of moisture, microbiological deterioration, textural changes and loss of nutrients and flavor which affect their quality (37). Furthermore, fresh mushrooms have a significant moisture content ranging from 85 to 95% and a rapid respiration rate (640.8 mg kg^−1^ h^−1^) at 10°C for *Lentinus edodes* and (200 to 500 mg kg^−1^ h^−1^) for *Agaricus bisporus* which can encourage microbial growth and enzymatic browning, thus accelerating the aging process (38). However, the factors responsible for the short shelf life of shiitake include moisture loss, color deterioration/ browning, modifications in flavor/aroma, texture, inherent nutritional and biochemical constituents, physical damage, respiration, and microbial attack (Figure 1).

### 3.1 Organoleptic quality

During the postharvest period, mushrooms undergo a rapid reduction in firmness, resulting in a reduced shelf life and heightened vulnerability to microbial contamination (39). Mushroom postharvest texture is influenced by factors such as water loss, wounds, mechanical injuries, and heat treatments (40). In the case of many fruits and vegetables, thermal processes result in the breakdown of cell-to-cell connections in structural molecules, along with membrane destruction and turgor loss, ultimately causing tissue softening. Shiitake firmness undergoes a rapid decrease post-harvest, declining from 17.3 N to approximately 13 N after 16 days of cold storage at 4°C (41). In mushroom texture evaluation, “Newton” (N) is used to measure the force needed to compress or puncture the mushroom, indicating its firmness and potential changes in texture or quality. Unlike other fruits and vegetables, mushrooms lack pectin structure. The cell wall of mushrooms primarily comprises glucans, chitin, and protein. Through heat treatment, chitin and the β-1,4-acetyl-glucosamine homopolymer create a rigid microfibril structure, strengthening mushrooms’ cell wall (42). Therefore, the drying process led to an increase in mushroom firmness and chewiness (43).

### 3.2 Loss of moisture

Freshly harvested shiitake mushrooms can contain up to 90% moisture (on a wet basis), and the decrease in moisture content during preservation plays a pivotal role in determining the overall quality of the fresh mushroom (43). Moisture content diminishes further in post-harvest storage because of cell damage and internal water transfer in mushrooms. This physiological process accelerates mushroom quality degradation, leading to shrinkage and weight loss. For instance, weight reductions of mushrooms stored at 4°C for 2, 7, and 12 days were documented as 0.07, 0.27, and 0.49%, respectively (17). Mushrooms are considered spoiled when water loss reaches 5% of their original fresh mass (44). Several factors cause the sudden elevation of cellular contents, malondialdehyde (MDA) levels, and changes in enzyme activity, such as peroxidase (POD), catalase (CAT), and superoxide dismutase (SOD). *Pl. Eryngii* mushrooms are considered spoiled and unsuitable for consumption if their weight loss exceeds 3.41% of their original fresh weight (45). To prolong mushroom freshness, it is essential to manage water loss at a specific low level. This can be achieved through methods such as packaging (46) or treatment with electrolyzed water (17).

### 3.3 Discoloration

Mushrooms are susceptible to browning due to microbial contamination or enzymatic activities, with enzyme activities considered the primary cause of browning on fruits and vegetables (47). In enzymatic reactions, phenolic substances undergo oxidation to form quinones, which are subsequently transformed into melanin, resulting in brown appearance on products (48). The presence of hydrogen peroxide activates phenol peroxidase, and in the presence of oxygen, polyphenol oxidase becomes active. These two factors cause surface browning of mushrooms (48). Hydrogen peroxide content is low in mushrooms. Polyphenol oxidase (PPO) is identified as the primary factor influencing postharvest browning in *Agaricus bisporus* (49). The rupture of cell membranes within the tissue results in the combination of polyphenol substrates with polyphenol peroxidases, initiating browning reactions. Typically, polyphenol oxidase (PPO) catalyzes the reaction of phenolic compounds in two stages: first, the hydroxylation of monophenols into diphenols, and second, the oxidation of phenolic substances into quinones (48). Tyrosinase, within the PPO family, is the enzyme accountable for the browning of *Agaricus bisporus* due to its elevated presence in mushrooms (50).

### 3.4 Nutritional content

Mushrooms possess a high protein content in their dry matter, comprising nine essential amino acids; furthermore, they are low in fat and exhibit a relatively elevated level of carbohydrates and fiber, with the presence of fat-soluble vitamins and ergosterol making mushrooms the exclusive vegetarian source of vitamin D (51). According to Valverde et al. (52), *Agaricus bisporus* contains 14.1% protein, 2.2% fat, 9.7% ash, and 74% carbohydrates. For every 100 g of fatty acids, the composition includes 77.7 g linoleic, 11.9 g palmitic, 3.1 g stearic, 1.1 g oleic, and 0.1 g linolenic. Chitin, glycogen, mannitol, and trehalose are components present in mushrooms’ carbohydrates (53). The total phenolic compounds in mushrooms exhibited a continuous decline from 500 to 400 × 10^−3^ g kg^−1^ during a 48-h storage period at 4°C with 85% relative humidity in *Agaricus bisporus*. Mushrooms exhibit distinctive aroma and flavor profiles, encompassing a range of volatile and non-volatile components, including soluble sugars, free amino acids, organic acids, polyols, 5-nucleotides, and monosodium glutamate (MSG) (54). Mushroom flavors are influenced by various processes. For example, the dosage of gamma irradiation applied had an impact on the quantity of total volatile compounds in food products (55).

## 4 Factors influencing storage quality of shiitake mushroom

Throughout the post-harvest phase, mushroom quality is closely linked to both internal factors (such as water activity, microbial activity, and respiration rate) and external factors (including mechanical damage, storage temperature and relative humidity). Comprehending the impact of these factors offers valuable insights into preserving mushroom quality.

### 4.1 Water activity

Water activity refers to the ratio between the equilibrium water vapor pressure of a given food item and the saturated vapor pressure of pure water at the corresponding temperature. This parameter significantly affects mushroom quality (56). Based on the concentration of free water, this factor is frequently regarded as a key determinant in processes leading to quality degradation. These processes encompass reactions like lipid oxidation, microbial stability, enzymatic and non-enzymatic activities, as well as alterations in the texture profile. Naturally elevated water activity (AW) in fresh mushrooms creates an optimal environment conducive to microorganism proliferation (57). According to Jaworska et al. (58) the water activities for air-dried blanched mushrooms, air-dried unblanched mushrooms, freeze-dried blanched and freeze-dried unblanched mushrooms were recorded as 0.298 ± 0.001%, 0.228 ± 0.004%, 0.041 ± 0.002% and 0.029 ± 0.003%, respectively. Mushrooms treated with 1% glycerol exhibited the lowest water activity (aw) compared to those treated with 0.1% potassium metabisulphite, 0.2% potassium metabisulphite, 1% CaCl_2_, 2% CaCl_2_, and 0.5% glycerol. Additionally, mushrooms with the lowest water activity demonstrated superior performance in retaining mushroom color, controlling microbial counts, enhancing dehydration ratio, improving rehydration ratio, and preserving crude fat (59).

### 4.2 Respiration rate

The post-harvest condition of edible mushrooms is notably influenced by their respiration rate, as the availability of oxygen in the surrounding air directly impacts their metabolic and respiratory activity, as evidenced by Abdelshafy et al. (60). To extend the storage period of edible mushrooms it becomes essential to maintain an ideal respiration rate highlighted in the research by Qu et al. (61) and Li et al. (62). The temperature and duration of mushroom preservation significantly influence the pace of this respiration process, as discussed in findings by Azevedo et al. (63). Due to their permeable and delicate cuticular composition edible mushrooms release a substantial amount of CO₂ h^−1^ with approximately 132–158 mL CO₂ kg^−1^ and 20–35 mL CO₂ kg^−1^ being expelled at 20°C and 5°C respectively, as reported by Wang et al. (64). When mushrooms are placed in storage after harvest, they undergo microbial degradation. This promotes increased oxygen consumption, water loss, browning, and microbial growth. This degradation occurs due to physiological stress induced by microbial activity or pest infestations, as highlighted by Meitha et al. (65). During the post-harvest preservation phase, mushrooms endure abiotic stress, hindering electron transport in their mitochondria and leading to heightened production of reactive oxygen species, as noted by Meitha et al. (65). Oxidative stress occurs when reactive oxygen species exceed cell antioxidant capacity. This type of stress damages cellular structures, resulting in the degradation of DNA, proteins, lipids and membranes as indicated by (66, 67). Typically, changes in respiratory metabolism directly impact mitochondrial membrane enzymes involved in respiration, such as succinate dehydrogenase and cytochrome C oxidase. This is highlighted by Li et al. (68).

### 4.3 Temperature and relative humidity

Mushroom postharvest quality is significantly influenced by storage temperature, as many biochemical, and microbiological processes associated with quality deterioration, such as respiration, color changes, and ripening, are closely tied to temperature (43). Generally, elevating the storage temperature speeds up mushroom aging, browning, weight reduction, and texture softening. Storing mushrooms at ambient temperature (20 to 25°C) for 1 day could lead to a decline in quality, resulting in issues like cap opening, discoloration, stem elongation, and texture softening (42). Ambient temperature, which is the average temperature of the surrounding environment or immediate surroundings, plays a crucial role in influencing the quality and shelf life of mushrooms during storage. When the storage temperature increased from 2 to 18°C, the transpiration rate of fresh oyster mushrooms increased from 1.82 (±0.20) to 3.88 (±0.41) g/kg at 86% relative humidity (42). A low and consistent temperature is maintained (above 0°C) for postharvest storage of mushrooms, as storing them below 0°C can lead to chilling injury (40). Maintaining high relative humidity (85–95%) during postharvest storage significantly reduces mushrooms’ transpiration rate, minimizing water loss and preserving their mass (43). According to Azevedo et al. (63), oyster mushrooms exhibited a minimal mass loss of 8.35% of their fresh weight when stored under vapor-saturated conditions at 2°C for 136 h (Table 2).

**Table 2:** Illustrates the impact of temperature and relative humidity on the shelf life of mushrooms.

Common name of mushrooms	Storage temperature	Relative humidity (%)	Shelf life (days)	References
Shiitake	16°C	˃90%	14 to 18 days	(60)
Oyster	0 to 5°C	95%	7 to 14 days	(41)
Straw	15°C	95%	6 days	(69)
White button	4°C	95%	22 days	(70)
Reishi/Manentake	0 to 4°C	85 to 90%	Several months	(71)
Milky	2°C	90%	8 to 11 days	(72)

### 4.4 Microbial infection

**Figure 2:**
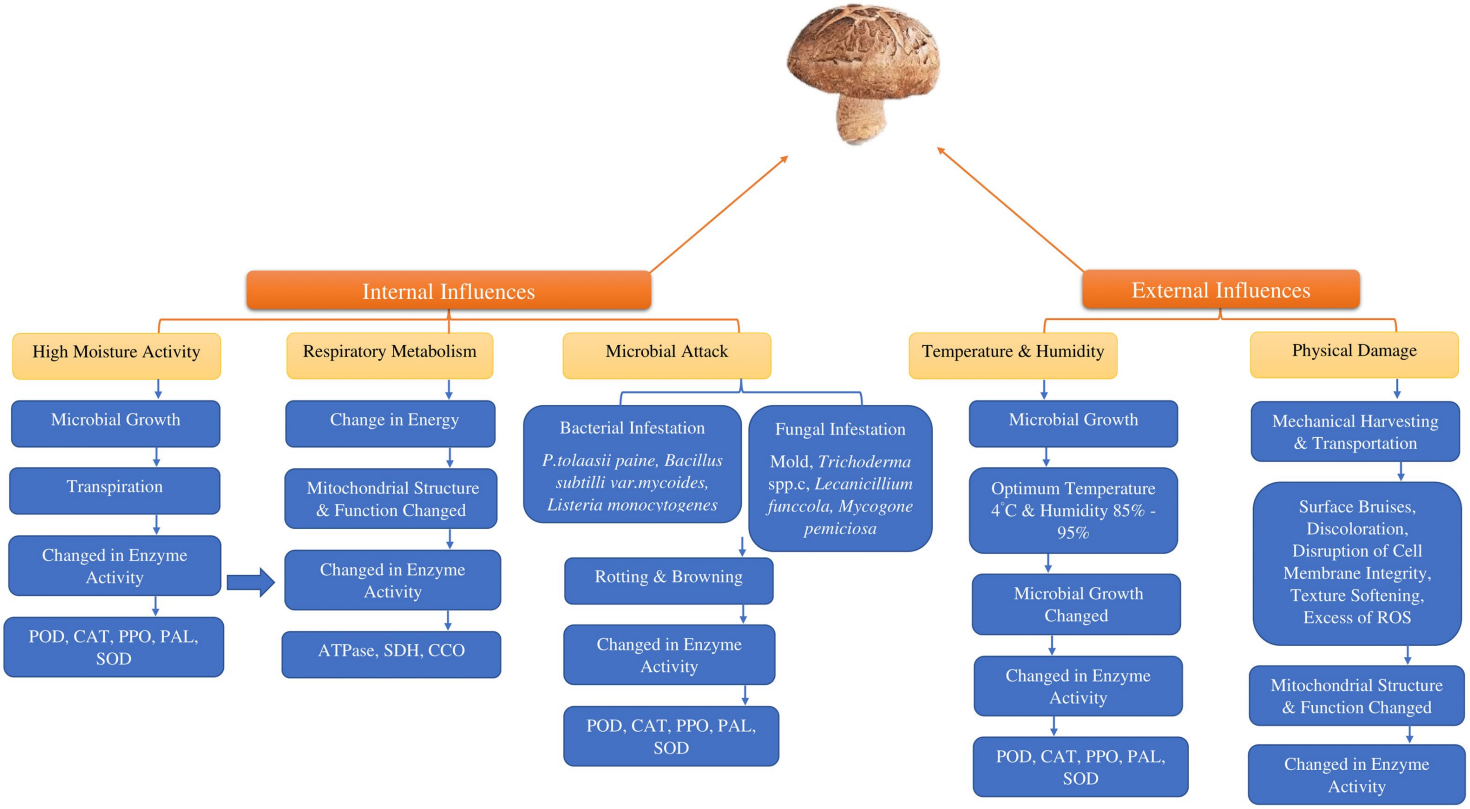
Factors influencing the post-harvest degradation of consumable mushrooms. (POD; peroxidase, CAT; catalase PPO; polyphenol oxidase, PAL; L-phenylalanine ammonia–lyase, SOD; superoxide dismutase, SDH; succinate dehydrogenase, CCO; cytochrome C oxidase, ROS; reactive oxygen species).

The absence of protective outer layers and the high-water content in edible mushrooms render them susceptible to various microorganisms during the post-harvest phase, resulting in a variety of changes in enzymatic processes (Figure 2). Some pathogenic bacteria that have an impact on edible mushrooms are *Listeria monocytogenes* (73–75), *Bacillus subtili* (73, 74) *Pseudomonas fluoresens* (76–79) and *Pseudomonas tolaasii* (79, 80). Furthermore, another type of microorganism, mold, can cause infections in edible mushrooms (81, 82). The detrimental effects of fungi-caused infections like *Trichoderma* spp., *Mycogone perniciosa*, *Lecanicillium funccola* and *Cladobotryum* spp., limiting agricultural output could lead to reduced crop yields and can be attributed to various factors (83, 84). Catalase and peroxidase enzymes are activated by microbial presence or insect infestations. This results in physiological disruptions that lead to undesirable changes in plant structural integrity.

## 5 Methods to preserve mushroom

### 5.1 Thermal process

**Figure 3:**
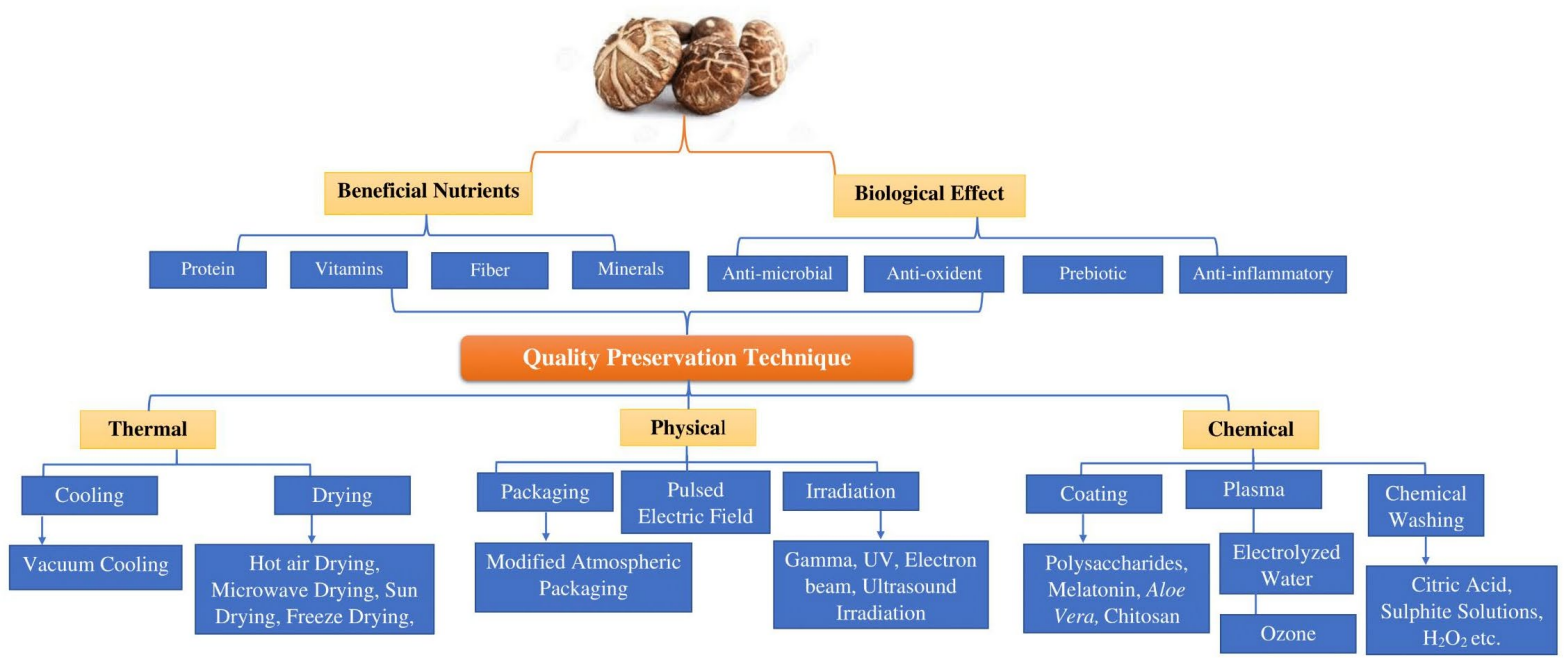
Illustrates the advantageous nutrients, physiological impacts, and approaches that influence the quality of shiitake mushroom (*Lentinula edodes*).

Post-harvest techniques can be classified into three categories: thermal, chemical, and physical. In Figure 3, we present a summary of the methods those have been investigated to decelerate or prevent chemical degradation and the proliferation of microorganisms while preventing recontamination of the mushrooms. Non-thermal drying of Shiitake mushrooms preserves color and structure but lacks aroma development, while thermal drying, especially with infrared hot air convection drying, offers a quicker process, delightful fragrance, and superior qualities like reduced oxidation and high polysaccharide content. Relative humidity drying minimizes shrinkage, and polysaccharides from infrared drying exhibit the highest antioxidant properties among tested methods (85).

#### 5.1.1 Drying

To achieve microbiological and physicochemical stability, drying is a widely employed food preservation technique. It operates on the principle of regulating water activity to a specified level. This method has a long history of prolonging freshness for food products (86). The current scalding method, which is carried out by the sample coming into direct contact with a medium like hot water or steam can significantly (*p* < 0.05) reduce the overall quantity of bacteria, increase drying effectiveness, and lessen the sample’s degree of browning during drying (87). But there have been reports of problems blanching with water and steam, which can result in nutrient loss such as protein, polysaccharides and vitamins (88). Crude protein content was lowered by 7.7% when using the solar drying method with a 5% infiltration concentration. However, crude fat, crude fiber, total ash, and carbs were all preserved at the same levels. Solar drying with a 5% infiltration decreased moisture by 7.7% while preserving the approximate composition of crude fat (2.3%), crude protein (25.1%), crude fiber (10.3%) total ash (10.2%), and carbs (44.4%). A microwave hot-air flow rolling dry-blanching method (MARDB) improves the drying effectiveness and quality of king oyster mushrooms. When drying and hot rinsing, microwaves can change the microstructure, which may impact drying properties, water status, and migration (89). The quality parameters, including color, water content, and polysaccharide content, were all greatly enhanced by MARDB. This also reduced drying time and totally inactivated PPO and POD. Excessive heat blanching decelerates the drying process while simultaneously reducing polysaccharides and phenolic compounds, resulting in decreased whiteness (90). Kurata et al. (91) used microwave vacuum dehydration (MVD) to dry shiitake mushrooms at varying microwave powers (25 W/g, 50 W/g, and 75 W/g of dry matter) and various absolute pressures (10 kPa,20 kPa,30 kPa,). Freeze drying (FD) achieves high-quality products by sublimating water directly from the solid to vapor phase, maintaining heat-sensitive properties like vitamins due to the low drying temperature (92). Freeze-dried products exhibit a porous structure due to ice crystal sublimation, but the process is hindered by high capital and energy costs from vacuum and refrigeration systems, resulting in limited throughput and slow drying (93). In summary, drying is a frequently employed food preservation method, which controls water activity to maintain microbiological and physicochemical stability (Table 3).

**Table 3:** Different drying methods modified atmosphere packing, irradiation condition, washing process, plasma, essential oils, plant extract and application of melatonin utilized for the preservation control of shiitake mushroom.

Methods	Processing	Results	Effect on the quality of storage	References
Hot air drying (HAD) Microwave HAD	Temperature 60°C	HAD exhibited the highest sulfuric aromatic compound percentage (7.7%), superior rehydration ability, and the least cap color variation, while MHAD had the highest polysaccharide content (240.28 mg/100 g) and notable percentages of ketones (4.22%), nitric aroma (1.17%) and aldehydes (0.26%)	It also impacts rehydration, but it yields greater and pleasant aroma and polysaccharides	(94)
Hot air drying	Temperature 65°C	Volatile compounds are released during different drying stages, resulting in a 66.48% reduction in size and a ΔE of 7.81 for fully dried mushrooms, indicating minimal color variation. (ΔE in colorimetry quantifies color differences, with higher values indicating more significant distinctions and lower values denoting greater similarity)	The initial drying phases emitted a subtle garlic odor, followed by a pronounced rotten egg scent during the middle stages, and finally a sautéed aroma in the late stages, attributable to varying concentrations of sulfur, alcohols, and carbon compounds.	(95)
Freeze drying (FD)	Temperature − 35 to-40°C	Maximum preservation of aromatic compounds.	Freeze-drying proved superior for mushroom storage	(96)
Sun drying	3 days with 12 h daylights Temperature 60°C. A period of 3 days with a total of 36 h of daylight, Temperature 26°C,	Elevated vitamin D levels and greater phenolic content compared to HAD.	The lowest total antioxidant capacity yield.	(97, 98)
Spraying FD	Temperature-25°C for 30 h	When compared to the control group, the detected volatile compounds ranged in recovery from 30.9 to 82.9%.	The detectability of volatile aromatic constituents within dehydrated particles was enhanced.	(99)
Treatment with O₃ (ozone)	The optimal O₃ concentration is at 3.21 mg/mᵌ.	Stored under a temperature of 4°C and a relative humidity of 90 ± 5% for duration of 14 days.	Reducing surface oxidation helps retain proteins and phenolic compounds while leading to a decrease in free amino acids	(100)
Washing with chemicals	H₂O₂, citric acid, and sodium metasulphite	After washing, Dried	The quality will remain unchanged.	(101)
Fumigation	Cinnamaldehyde 40 nmol/L with ethanol 30%	After treatment, the preserved temperature was maintained at 4°C for duration of 30 days.	Enhanced stress resilience, sustenance of turgidity, and heightened antioxidant efficacy.	(102)
MAP (Modified atmosphere packaging)	High CO₂ Packaging (O₂ 15% + CO₂ 20%) Low CO₂ Packaging (CO₂ 2% + O₂30%) High N₂ Packaging (O₂ 15% + N₂ 85%)	Storage maintained at a temperature of 4°C for a period of 10 days, RH. 90%	Ideal processing conditions involve elevated total phenolic content, a gas mixture of 20% CO_2_ and 15% oxygen, and a gradual reduction in color darkening.	(103)
Coating	Guargum (5, 15, and 25%) + chitosan (1%), Coated at 20°C for 2 min enclosed within Polyethylene bags	Stored for duration of 16 days at a temperature of 4°C with a relative humidity ranging between 85 to 90%.	Maintained mushroom texture and sensory attributes, prevented deterioration, and safeguarded components of cell wall	(104)
Gamma radiation	1.0 to 2.0 kGy	Stored at a temperature of 4°C with a relative humidity (RH) of 90% for a duration of 20 days.	Mushrooms treated with 1.0 kGy exhibited improved color and appearance while preserving their moisture content. However, irradiation at 2.0 kGy resulted in undesired tissue fluid leakage and a deteriorated appearance.	(105)
Pulsed light	Quantity of light 68.mj/cm^2^, Pulse number; 3	Storage under room temperature conditions in an oxygen (O₂) environment, with the temperature set at 4°C within the oxygen atmosphere.	There has been a notable rise in caffeine levels and gallic acid, along with increased emissions of volatile substances, particularly those with 8-carbon structures.	(106)
Cellulose + ultrasound	Ultrasound administered at a rate of 0.21w/cm^2^ in conjunction with cellulose treatment under conditions of 60°C temperature. The cellulose solution is prepared at a concentration of 0.2%, with a solution pH of 4.5	Samples were frozen and kept in storage before undergoing analysis.	Enhanced water mobility and elevated levels of phenolic compounds.	(107)
UV-A UV-B	Dose: 23,35.3 and 25.2kj/ m^2^ Dose: 25,50 and 75 kj/ m^2^	Before analysis, samples were freeze dried and stored.	UV-A exhibited lower content of vitamin D₂ as compared to UV-B. D₂ content increased with increasing the radiation in mushroom gills	(108, 109)
UV-C	Exposure dosage: 4kj/m^2^, enclosed within polyethylene bags	The sealed bags were placed in storage at a temperature of 1°C with a relative humidity of 90% after a three-day period.	Low-temperature storage-maintained tissue solidity and resulted in higher levels of flavonoids and ascorbic acid, enhancing antioxidant properties.	(56)
Lemon essential oil (LEO)	Various concentrations of essential oil were employed, ranging from 0, 3, 6, 9 and 12%, while maintaining storage conditions at 4°C and a relative humidity of 90%.	Storage duration of 12 days	Preventing bacterial growth and mitigating the aging and quality degradation of mushrooms.	(110)
*Cinnamon* essential oil	Paper treated with different combinations: Uncoated paper, Paper coated with equal parts of starch and microcapsules, Paper coated with a starch-to-microcapsule ratio of 1:3, Paper coated with a starch-to-microcapsule ratio of 1:5, and CEO-coated paper.	Storage duration: 4 days, Storage conditions: Maintained at a temperature of 4°C	The combination of starch and microcapsules (in a 1:5 ratio) applied to CEO-coated paper has a preserving effect, helping to reduce the aging and quality deterioration of mushrooms	(111)
Essential oil of cumin	Storage temperature 4°C, Subjected to Cumin essential oil treatment, As well as the application of CEO combined with chitosan nanoparticles.	Storage duration of 20 days.	Inhibiting microbial growth, retaining desirable texture, sustaining an appealing appearance, and exhibiting strong antioxidant capacity.	(112)
Cold plasma	Power of Plasma: 650 W. Drying temperature is 50,60 and 70°C	Mushroom treated with plasma depicted high moisture diffusivity, increase in flavonoids, phenolics and antioxidant activity	Direct plasma showed better moisture migration, which improved drying rate and increased phenolic and flavonoid content.	(113)
Edible coating	Polysaccharides	The coating decreases the respiration of the mushroom, reduces weight loss, increases stiffness, prevents change in color, and slow down the mushroom’s texture deterioration.	The coating, rich in ascorbic acid, retards phenolic compound loss, prevents mushroom proteins and carbohydrates from degradation, and sustains high enzymatic activity.	(39)
Edible coating of *Oudemansiella radicata* water soluble polysaccharide (ORWP)	Storage temperature 4°C, R.H. 85% _95%, ORWP5:5,10,15,20 gL¯^1^ coating, Control washed with water no coating,	Storage time 18 days	Reducing the aging process and mushroom quality decline, High antioxidant activity, Hyperactivate enzymes	(39)
Melatonin coating (A600605-0005)	Storage temperature 4°C, Immersed in 0.05, 0.1 and 0.2 mM melatonin Control treatment	Storage times 12 days	Delay aging, Maintain quality and damp electron leakage	(114)
Melatonin coating	Control treatment, Cd2(2 μM CdCl₂) Cd5(5 μM CdCl₂) Cd8(8 μM CdCl₂) Melatonin 50μΜ MT50 solution, 100 μM MT100 solution, 200 μM MT200 solution, Cd + MT treated	Storage times 12 days	Improve antioxidant activity, Maintain high nutritional value, ROS reduced	(115)
Immersed for 2 h *Solanum lycopersicum* (Tomato)	MT 100 μmol/L	Storage temperature 4°C	Controlling the arginine pathway and increasing the expression of SIZAT2/6/12 genes, which are associated with the C-repeat binding factor (CBF) gene	(116)
Immersed for 2 h *Cacumis sativus* L. (Cucumber)	MT 200 μmol/L	Storage temperature 8°C	Elevating polyamines and abscisic acid (ABA) levels through the up regulation of csZat12 transcription.	(117)
Immersed for 2 h *Fragaria anannasa* (Strawberry)	MT 0, 1, 10, 100, 1,000 μmol/L Optimum 100	Storage temperature 4°C	Increased total phenolic & flavonoids, Increase in endogenous melatonin Increase in antioxidant activity, GABA content, accumulation of H_2_O_2_ triggered	(118, 119)
Saprayed *Solanum tuberosum* (Potato)	MT 0, 1,000, 3,000, 6,000, 8,000, 10,000 μmol/L Optimum 10,000	–	Decrease in potato late blight	(120)

#### 5.1.2 Sun drying and Hot air drying

**Figure 4:**
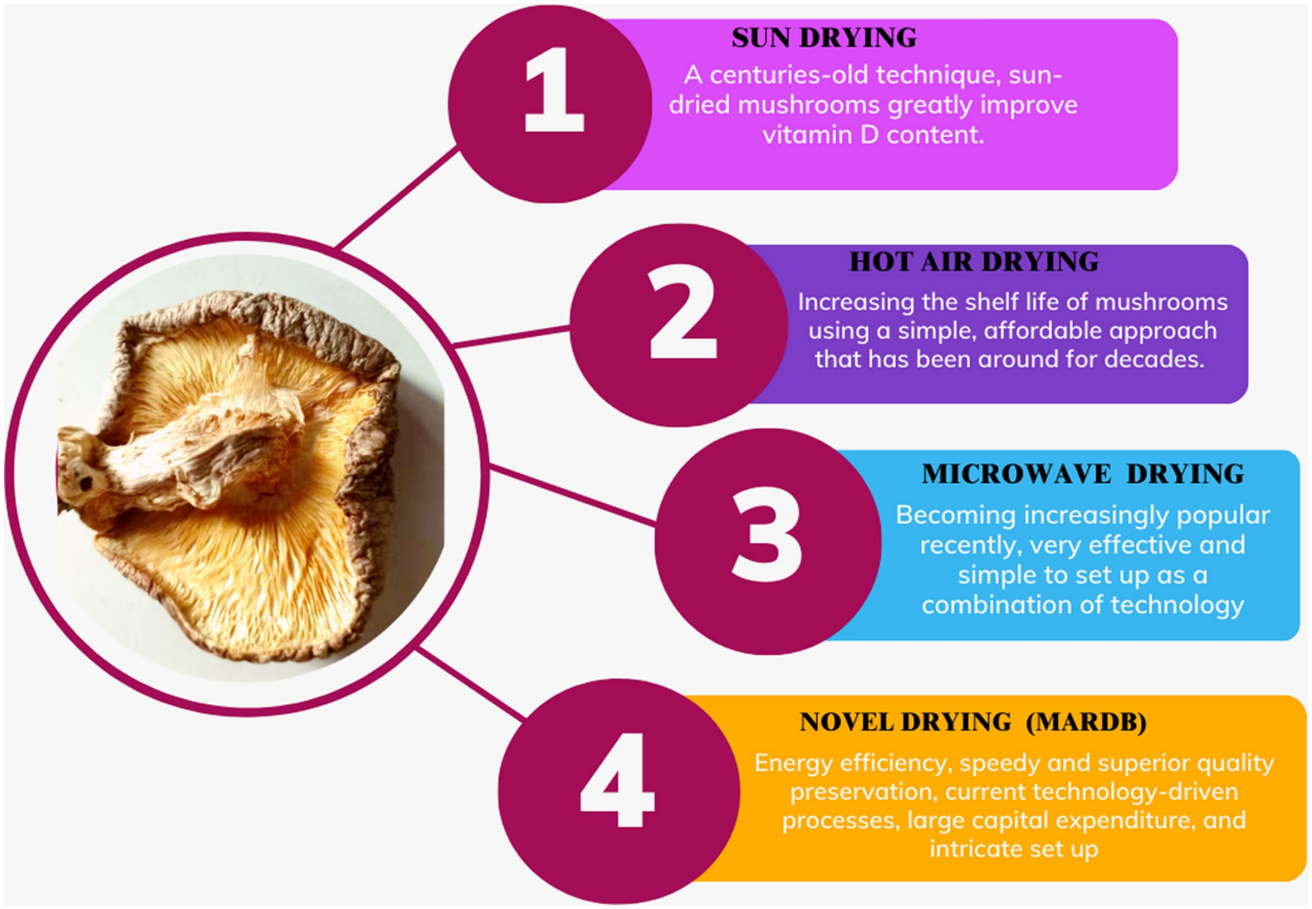
Post-harvest handling of shiitake mushrooms involves the utilization of both traditional and contemporary thermal drying methods.

Mushrooms have been preserved by the sun for many years, which is one of the most popular methods for food preservation. Mushrooms are immediately sun-dried after harvest, continuing until they reach the desired moisture level of 10 to 13% (121). Sun drying, despite being weather-dependent and slower with inferior drying efficiency compared to high air velocity drying (HAD), was previously adequate for preserving mushrooms effectively. Sun drying may take as long as 3 days for each iteration to attain thorough desiccation, whereas hot air drying (HAD) only requires 12 h per cycle at a temperature of 60°C (97). Shiitake mushrooms that have been sun-dried exhibit higher concentrations of vitamin D2 than mushrooms dried using alternative methods, primarily due to the sun’s facilitation of ergosterol conversion into vitamin D₂ (122). The cumulative phenolic content in shiitake mushrooms dried in the sun exceeds that of mushrooms dried through the hot air drying (HAD) method according to Kim et al. (97). Shiitake mushrooms that were sun-dried indoors at 26°C for 36 h exhibited reduced levels of total phenolic content (93.4 to 3.9 mg/100 g in fresh shiitake), total antioxidants (109.5 to 5.4 mg/100 g in fresh shiitake), and scavenging activity (43.2 to 6.6%) in comparison to microwave and freeze-drying techniques (98). Conversely, hot air-drying employs controlled elevated temperatures typically ranging from 50°C to 80°C (122°F to 176°F), ensuring faster, consistent, and precisely regulated drying, making it ideal for large-scale commercial operations where temperature control is crucial for efficient drying (97) (Figure 4).

#### 5.1.3 Cool storage

Mushroom shelf life must be extended by quickly removing heat after harvest and maintaining a low storage temperature. Low temperature is useful for minimizing moisture loss from mushrooms, decreasing microbe growth, and limiting their respiration rate (123). The browning was reduced by 75% by lowering the storage temperature from 25°C to 3°C (124). Research on *Flammulina velutipes* fruiting bodies examined the impact of refrigeration on nutritional content and active ingredients, revealing a significant decline in the natural antioxidant L-ergothioneine concentration after 8 days at 5°C in both bright and dark conditions, while total phenolic content increased notably over 10 days of refrigeration under fluorescent lighting; however, the antioxidant capacity decreased after storage in both dark and light conditions for five and 8 days (125). Vacuum cooling, despite its higher initial cost than conventional cooling systems, is the natural antioxidant L-ergothioneine concentration after 8 days at 5°C in both light and microbial growth (126). Mittal et al. (127) explored the impact of various pre-cooling methods and packaging materials (room cooling, hydrocooling, vacuum cooling, air forced cooling, polypropylene, polyvinylchloride and high impact polystyrene + polyvinylchloride) on the shelf life of *Agaricus bisporus* stored in specific environmental conditions (14 to 16°C and relative humidity 56 to 83%), revealing that vacuum cooling was the most effective pre-cooling method, followed by forced air cooling and room cooling, while punnet packages (HIPS, PP and PVC) were the preferred packaging materials, extending the shelf life and preserving the quality of mushrooms for up to 4 days. According to Tao et al. (128) vacuum cooling treatment and various storage conditions, including cold rooms and modified atmosphere packaging, affected antioxidant enzyme activity in *Agaricus bisporus* mushrooms, revealing that vacuum-cooled mushrooms exhibited enhanced activity of antioxidant enzymes like superoxide dismutase, catalase, peroxidase and polyphenoloxidase along with a reduction in malondialdehyde levels and the generation of superoxide anions. The most significant activation of the enzymatic antioxidant system was detected in mushrooms stored using modified atmosphere packaging combined with vacuum cooling treatment. In short, to extend the storage life of mushrooms, the use of lower storage temperatures can help minimize moisture loss and microbial growth. However, it may impact nutritional quality. Vacuum cooling, when paired with suitable packaging materials proves to be a successful approach for increasing shelf life. The combination of vacuum cooling and modified atmosphere packaging enhances antioxidant enzyme activity and lowers oxidative stress (128).

### 5.2 The physical process

Physical methods like modified packaging with controlled atmospheres and ozone treatment effectively preserve mushrooms by slowing deterioration and promoting microbial growth. Irradiation controls pests and pathogens, ensuring prolonged freshness and visual appeal.

#### 5.2.1 Packaging

When mushrooms are stored for 28 days at 4°C post-harvest using active packaging infused with zeolite and acai extract they may experience alterations in their typical physical and chemical characteristics. Notably, mushrooms kept in these enhanced containers exhibited elevated levels of vitamin C and enhanced antioxidant potency, with a more pronounced effect observed in containers containing zeolite (129). For optimal color, maintain a moisture level between 87 and 90%. The most suitable humidity level for packaging mushrooms is 96% according to Wakchaure (130). Packaging techniques create a controlled environment for food products, preventing quality degradation and extending shelf life. Two primary methods include using plastic bags, allowing gas exchange through packaging films, and preserving items at a low temperature. This is done under consistent environmental conditions. Modified atmospheric packaging, characterized by higher CO₂ and lower O₂ levels compared to air, has been found to reduce respiration rates, ethylene production, sensitivity, microbial spoilage, and induce changes in physiological and biochemical processes.

However, without proper storage conditions, glycosaminoglycans and polysaccharides may diminish. An alternative approach known as Modified Active Packaging (MAP) has been devised to combat surface browning triggered by enzymatic reactions and anaerobic microbial attacks, by maintaining oxygen levels higher than carbon dioxide. According to Li et al. (131) Shiitake mushrooms were enclosed in three distinct packaging environments with varying oxygen levels: HOP (high oxygen packaging) with an initial oxygen concentration of 100%, MOP (medium oxygen packaging) with an initial oxygen level of 50%, and LOP (low oxygen packaging) with an initial oxygen content of 35% and an initial CO₂ content of 5%. During a seven-day storage period at 10°C and 90% relative humidity, an identical package filled with ordinary air served as the control. While all packages experienced a decrease in polysaccharides, color and integrity were preserved. Notably, LOP and AIR released more ethanol, while MOP and HOP released less. Additionally, the active packaging resulted in higher total amino acid and phenolic content than the control group. Wan-Mohtar et al. (103) explored the effectiveness of high CO₂ packaging (HCP) with a gas composition of 15% O₂ and 20% CO₂ in preserving king oyster mushrooms quality. In comparison to both the control group and low CO₂ packaging with 30% O₂ (HOP) and 2% CO₂ (LCP) it exhibited better outcomes. It displayed the highest cumulative phenolic content and demonstrated superior effectiveness in preserving color and aroma of *P. eryngii*.

#### 5.2.2 Irradiation process

The adoption of advanced post-harvest techniques, such as food irradiation, has the potential to enhance accessibility and prolong storage durations, and this technology has gained widespread acceptance. Extensive research has been conducted to assess the nutrient safety and technological benefits of irradiated food (132). This approach to mushroom preservation is safe and environmentally sustainable. Irradiation preserves the flavor, color, nutritional content and enhancing the flavor of mushrooms without leaving behind any detrimental residues. However, the influence of irradiation on mushrooms’ nutritional content can differ based on elements such as the specific mushroom variety the use of additional preservation techniques, radiation dosage, and the type of radiation source employed, which may include UV radiation, electron beams and gamma radiation (133).

UV radiation, in particular, enhances the vitamin D2 content in mushrooms, eliminates spoilage-causing microorganisms, and enhances the functional attributes of food. While doses of radiation 1–2 kGy can effectively eliminate insects, the eradication of bacteria may necessitate higher radiation doses of up to 10 kGy (134). WHO (world health organization) regards any food item exposed to radiation doses up to 10 kGy as safe and permissible (135). According to Fernandes et al. (133) gamma and electron beam irradiation have been found to delay or inhibit microbial growth, prevent stem elongation, reduce surface browning, and slow down mushroom maturation. This results in an extended shelf life for mushrooms, with the duration ranging from 10 to 15 days depending on the type of mushroom and the level of radiation exposure. Comparable results can be achieved through UV irradiation, with the added advantage of aiding the transformation of ergosterol found in shiitake mushrooms into Vitamin D2, thereby enhancing its nutritional value (136). Electromagnetic waves emanate from α and β rays originating from atomic nuclei. On an international scale, there are two officially sanctioned sources ^137^Cs and ^60^Co. Gamma radiation, by inhibiting cap expansion, stem elongation, discoloration, weight loss, and by enhancing the microbiological quality, prolongs the storage duration of mushrooms after harvest (137). Depending on the mushroom variety and the intensity of gamma radiation applied, irradiation produced varying impacts on the carbohydrates, protein, and micronutrients, including ash and tocopherols content in mushrooms (138). Electron beam irradiation, when compared to gamma radiation, offers a more convenient process with higher dosage rates and greater flexibility in starting and stopping. Exposure to radiation doses as high as 4 kGy showed enhancements the texture and color stability during preservation with sugars acting as the main source for mushroom respiration. Consequently, during preservation with high respiration rates often associated with increased degradation, a decrease in sugar levels is typically observed (139). Nevertheless, native dried mushrooms exposed to irradiation by electron beam demonstrated lower concentrations of acidic compounds when compared to their non-irradiated equivalents and electron beam irradiation resulted in an augmentation of the mushrooms’ antioxidant capability (140). In conclusion, active packaging, optimal moisture levels, and controlled atmosphere packaging methods enhance mushroom quality, while Modified Active Packaging and high CO₂ packaging combat browning and preserve quality. Food irradiation techniques, including UV radiation, electron beam irradiation, and gamma radiation, can modify mushroom characteristics, while ultrasound and pulsed light exposure have potential in preserving mushrooms. Table 3 provides a summary of the impact of UV, pulsed light, ultrasound, gamma irradiation and electron beam technologies on maintaining the freshness of fresh shiitake mushrooms.

### 5.3 Chemical treatment

Chemical preservation methods offer benefits in microbial control and shelf life, but the choice of cleaning chemicals requires careful consideration to avoid impacting mushroom quality. This is done by emphasizing the importance of thorough rinsing and drying with chemicals. Table 3 explains the consequences of chemical treatment on fresh shiitake mushrooms.

#### 5.3.1 Washing with anti-microbial agents

Freshly harvested shiitake mushrooms often carry soil microorganisms and other impurities, which can be effectively removed through a water rinse. However, using water alone, while it may increase surface moisture, can potentially accelerate microbial growth and spoilage. To address this concern, antimicrobial agents like citric acid, sodium metabisulfite, sodium chloride or sodium hypochlorite are introduced into the water during washing. Furthermore, agents that inhibit browning may be employed to reduce surface browning. The introduction of chlorine dioxide became essential after it was determined that sulfur salts had adverse impacts on the overall quality of mushrooms by Singh and Sindhu (101); Ramteke et al. (141) conducted a study on shiitake mushrooms that had undergone chemical washing and drying. They examined the effects of hydrogen peroxide, citric acid, and sodium metabisulfite on post-harvest shiitake mushrooms. The findings indicated minimal sensory quality variations in terms of flavor, color, and overall sensory attributes between treated and untreated mushrooms. In a separate study, shiitake mushrooms were wrapped in PVC films, subjected to a wash with water containing 200 mg/L of chlorine. They were then stored at different temperatures (7°C, 10°C, and 15°C) for 15 days. Although initial washing reduced microbial counts, subsequent storage led to faster browning and bacterial growth than untreated mushrooms. However, mushrooms washed with a solution containing oxine (50 ppm), calcium chloride (0.5%), and sodium erythorbate (0.1%) exhibited a significant reduction in microbial load and delayed color deterioration, as observed in Wakchaure (130). According to Guan et al. (13) pre-treating mushrooms with 3% hydrogen peroxide (H₂O₂) water prior to UV-C light application resulted in a more substantial reduction in microbial count (0.85 log CFU/g) and an increase in total phenolic and ascorbic acid content during 14 days of storage, proving most effective in preventing lesions and browning in the mushrooms.

#### 5.3.2 Fumigation

Fumigation is a common preservation technique used to retain agricultural products’ quality and increase shelf life. Essential oils are obtained from plants and are frequently used for fumigation because they perform a number of physiological processes, including antifungal, antibacterial, and antioxidant qualities. According to Jiang et al. (142) Freshly harvested shiitake mushrooms were fumigated with cinnamaldehyde, thyme, and clove essential oils at a concentration of 5 μL/L for 1.5 h at 10°C, followed by storage at 41°C and 90% relative humidity for 20 days. The evaluation of their antioxidant properties was carried out by measuring parameters such as H₂O₂ content, O₂ production rate and performing DPPH and ABTS radical scavenging assays. The results demonstrated a significant improvement in mushrooms’ antioxidant activities after exposure to cinnamonaldehyde. (DPPH 2, 2-diphenyl-1-picrylhydrazyl, is a stable free radical commonly employed to assess antioxidant capacity. Its reaction with antioxidants results in a color change, allowing quantification of antioxidant activity. ABTS 2,2-azino-bis (3-ethylbenzothiazoline-6-sulfonic acid) is utilized in antioxidant assays and like DPPH undergoes a color change upon interacting with antioxidants, aiding in antioxidant capacity assessment). This method preserved mushrooms’ natural antioxidant properties but also extended their shelf life by protecting them from microbial deterioration. Furthermore, this fumigation technique effectively prevented sensory quality degradation observed during shiitake mushroom storage. In a separate investigation shiitake mushroom subjected to a 5 μL/L cinnamaldehyde treatment and stored at 4°C for 15 days displayed no indications of deterioration or weight loss. Additionally, there was a substantial reduction in bacterial proliferation and phenolic compounds remained constant (143). According to Qian et al. (144) Subjecting mushrooms to cinnamaldehyde fumigation at a concentration of 40 nmol/L dissolved in 30% ethanol and subsequently storing them at 4°C for 30 days had no effect on the overall quantity of total phenolic compounds. However, it improved their stress resistance and bolstered their antioxidant potential.

#### 5.3.3 Edible coating

Edible coatings have been extensively researched and created for mushroom post-harvest preservation. Coatings are a semipermeable barrier to CO₂, O₂, and moisture, changing the gaseous composition at the interface of the coating product. It can limit respiration, reduce moisture loss, delay ripening and maintain mushroom color and firmness. Chitosan, a naturally occurring biodegradable polymer, can serve as an edible covering to mitigate changes in mushrooms’ nutritional content while they are preserved (145). Liu et al. According to a study published in 2016, PA-g-CS (Protocatechuic acid grafted chitosan) combined with antioxidants showed promise for preserving king oyster mushrooms after harvest. The findings revealed that the PA-g-CS III coating, characterized by a high grafting rate, yielded the best outcomes by maintaining desirable textural attributes, minimizing membrane lipid peroxidation, elevating the activities of ascorbate peroxidase (APX), superoxide dismutase (SOD), catalase (CAT) and glutathione reductase (GR) while reducing the activities of polyphenol oxidase (PPO) (146). Shiitake mushrooms, when coated with a mixture of chitosan (1%) and guar gum (at concentrations of 5, 15, and 25%), and subsequently stored at 4°C with a relative humidity of 85 to 90% for 16 days, effectively preserved their firmness, prevented protein and ascorbic acid loss, increased the levels of soluble solids, sugars, and malondialdehyde, while preserving their flavor (104). The application of an *Aloe vera* coating enriched with basil oil at a concentration of 500 mL per liter to pristine mushrooms not only reduced the respiration rate but also maintained their firmness by preventing the differential leakage of electrolytes and the accumulation of malondialdehyde (147). Hence, the use of coatings and edible films enhances mushrooms’ antimicrobial properties and sensory attributes by mitigating respiration, weight loss, texture changes, and discoloration. In another study, shiitake mushrooms coated with a 1.5% w/v Nano-Ag film and stored at 4°C effectively reduced weight loss, prevented undesirable decay, surface discoloration, and other negative consequences throughout the 16-day storage period. Moreover, it effectively inhibited the proliferation of various microorganisms, including mesophilic, psychrophilic, yeast, molds, and others, thus preventing microbial spoilage (148).

#### 5.3.4 Ozone

Ozone is a strong antibacterial agent that is used to increase the extended durability of food owing to its elevated oxidizing potential causes it to quickly inactivate microorganisms by interacting with their cellular elements and intercellular enzymes. In a study conducted by Liu et al. (100) shiitake mushrooms were exposed to intermittent and single ozone treatments, then stored at a temperature of 4°C for a period of 14 days with 90% relative humidity demonstrating that periodic application at the ideal dosage of 3.2 mg/m^3^ outperformed the single treatment preserving proteins and phenolic compounds, inhibiting polyphenol oxidase activity to inhibit the occurrence of surface discoloration and decreasing the accumulation of unbound amino acids within the mushrooms. The study proposed that in order to substantially prolong the storage duration and boost the antioxidant capabilities of shiitake mushrooms, they should be subjected to ozone treatment for 30 min every 5 days applying a dosage of 3.2 mg per cubic meter (3.2 mg/m^3^). Yang et al. (149) observed similar results and found that utilizing ozone at a concentration of 4.28 mg/mᶾ, followed by preservation at 4°C for 25 days reduced nutrient loss and prevented surface expansion. The spoilage of mushrooms after harvest accelerates when no antimicrobial treatment is applied as the microbial load on the mushrooms increases during storage. Ozone, or triatomic oxygen, owing to its potent oxidizing properties exhibits robust antibacterial activity (150). According to Anjaly et al. (151) mushroom samples were exposed to ozone with different concentrations 10 and 15 ppm and durations 5 and 10 min. Subsequently, treated mushrooms were packaged in HDPE (high density polyethylene) material under both regular and vacuum atmospheric conditions and stored at 4°C with 90% relative humidity for a period of 15 days. Notably, the untreated mushrooms experienced a firmness increase from 3.5 to 14.1 g after 5 days of storage, while those treated with 10 ppm and 15 ppm of ozone exhibited the least firmness increase when stored in regular atmospheric conditions, as opposed to vacuum packaging. Zalewska et al. (152) explored mushroom samples were subjected to gaseous ozone treatment with different concentrations 0.05, 1.0, and 2.0 mg/L and exposure durations 30 and 60 min. The treated mushrooms packaged in PET (polyethylene terephthalate) boxes and wrapped with PVC (polyvinyl chloride) before being stored for 0 to 14 days at 2°C with 80% relative humidity. The firmness of untreated mushrooms increased from 15.3 to 19.6 N after 4 days and subsequently decreased to 10.95 N after 14 days. Mushrooms treated with 0.5 mg/L of ozone for 30 and 60 min showed firmness increases from 15.3 to 16.4 N and 18.1 N after 4 days, followed by declines to 5.9 N and 14.3 N after 14 days. In the case of ozone treatment at 1.0 mg/L for 30 min, the firmness increased from 15.3 to 17.7 N after 4 days and then decreased to 9.5 N after 14 days. However, after 60 min of ozone treatment, the firmness increased to 16.5 N from 9.6 N. With an ozone treatment of 2.0 mg/L for 30 and 60 min, the firmness increased from 15.3 to 17.5 N and 17.2 N after 14 days.

#### 5.3.5 Plasma and electrolyzed water

A burgeoning technology called cold plasma could supplant conventional storage techniques (153). When an electromagnetic field or electric field is applied to any gas, it undergoes ionization. This results in electrons colliding with the molecules or atoms within the gas and causing ionization. This, in turn, leads to the presence of chemically active entities like superoxide, free radicals, reactive oxygen species, and others within the plasma (154). These compounds inhibit various enzymes and bacteria while preserving the nutritional, physical, and sensory attributes of the materials intact (155). Xu et al. (156) examined the impact of plasma-activated water on postharvest quality of button mushrooms stored for 7 days at 20°C, revealing a reduction in bacterial and mold counts by 1.5 log and 0.5 log respectively, delayed firmness loss, and no significant alterations in color, pH, or antioxidant properties. Electrolyzed water generated through the electrolysis of a saline solution is a versatile disinfectant suitable for the food industry. Its effectiveness against microbes is determined by variables like the concentration of free available chlorine that forms hypochlorous acid (HClO) and its oxidation–reduction potential (ORP) by Rahman et al. (157). Aday (17) investigated the effect of electrolyzed water with different concentrations of 5, 25, 50 and 100 mg/L in combination with a passive modified atmosphere on button mushrooms. He revealed that those treated with 25 mg/L exhibited reduced browning, delayed weight loss and maintained texture. According to Castellanos-Reyes et al. (41), a low concentration of electrolyzed water with three other disinfectants aqueous ozone, 1% citric acid and sodium hypochlorite solution achieved reductions of 1.35, 1.08, and 1.90–2.16 log CFU/g in total aerobic bacteria, mold and foodborne pathogens respectively, following a 3-min treatment at room temperature (23 ± 2°C).

#### 5.3.6 Preservation of mushrooms using essential oils

Demand for high-quality edible mushrooms is rising globally. There is a growing focus on environmentally and economically sustainable preservation methods. This is due to the promising potential of botanical extracts and essential oils to enhance edible mushrooms’ shelf life due to their diverse chemical components.

I. Lemon & oregano essential oils.

Lemon essential oil, derived from citrus lemons, enhances food preservation and flavor due to its potent antioxidant and antimicrobial properties, demonstrating notable antibacterial and antioxidant capabilities when integrated into chitosan/zeaxanthin composite membranes at different concentrations (158). As a consequence, *Agaricus bisporus* aging was delayed, and postharvest quality was maintained by suppressing microbial growth and respiration (110). Among the mushrooms wrapped in films infused with 6% lemon essential oil, the lowest browning and respiration rates were observed. Furthermore, these mushrooms effectively preserved their antioxidant attributes, improved tissue density, and prevented browning, while also demonstrating strong inhibitory effects against foodborne bacteria such as *Staphylococcus aureus* and *Escherichia coli* (110). The primary constituent carvacrol in oregano essential oil, is a potent bioactive substance renowned for its antioxidative and antimicrobial properties (159). At a concentration of 0.25 mg/mL, oregano essential oil completely inhibited *S. aureus* growth and exhibits antifungal properties against *Candida albicans*. Carvacrol distinguished by its phenolic hydroxyl group is thought to function as a peroxide radical generator in the oxidation process. This interrupts the lipid peroxidation cascade and safeguards against the oxidation of lipids. This research revealed that when stored and preserved, edible mushrooms exhibited better effectiveness in preventing microbial intrusion at higher concentrations of oregano oil (160).

II. Cumin & Cinnamon essential oils.

Cumin is a versatile spice extensively utilized in culinary preparations and is the world’s second-most frequently employed spice trailing only behind black pepper (161). Its potent antimicrobial characteristics are also used to safeguard edible mushrooms’ longevity (162). When preserving *A. bisporus* at 4°C with chitosan nanoparticles as a delivery system for cumin oil, microbial presence was reduced compared to conventional packaging. This method also played a role in maintaining antioxidant potential, enhancing tissue firmness and preventing the formation of brown spots in Table 3 (112). Cinnamon oil, an organic volatile oil recognized for its strong antimicrobial attributes, is a more common choice for preserving edible mushrooms (111, 163). When used in appropriate quantities cinnamon oil can fumigate mushrooms, delaying cap formation and preventing mushroom aging. Cinnamaldehyde’s remarkable antibacterial properties are attributed to its ability to alter the arrangement of lipids within the membranes of harmful microorganisms, ultimately causing membrane disruption (164). By inhibiting the growth of *Salmonella typhimurium* and hindering the production of the ATP synthase alpha chain protein involved in ATP synthesis. It also prevents the division of *Bacillus cereus* into newly formed cells (165). Through the reduction of the droplet size to ensure even distribution within the packaging, it significantly slows down the aging of *A. bisporus*. This is when subjected to refrigerated storage. This can be attributed to cinnamaldehyde’s antibacterial and anti-oxidative properties (166). According to Nair et al. (167) developed hybrid nanofibers by combining maize alcohol-soluble protein and ethyl cellulose in different ratios, incorporating CEO into electrospun fibers for *A. bisporus*. This approach successfully retained moisture and antioxidant capacity, boosted antioxidant enzyme activity, minimized browning enzyme activity, postponed aging and extended *A. bisporus* shelf life. Pan et al. (168) prepared cross-linked electrospun polyvinyl alcohol/CEO/βcyclodextrin (CPVA-CEO-β-CD) nanofiber films using electrostatic spinning incorporating CEO in PVA and β-CD-based fibers to achieve a controlled release of antibacterial agents. This method inhibited both Gram-positive and Gram-negative bacteria, prolonging the product’s shelf life.

*III. Satureja khuzistanica* & Turmeric essential oil.

*Satureja khuzistanica* possesses antiviral, antifungal, and antibacterial properties, along with vasodilatory, antidiarrheal, antispasmodic, hypolipidemic, and antioxidant qualities (169). According to Nasiri et al. (15) the impact of baicalin gum (TG) coatings with different concentrations (100, 500, and 1,000 ppm) of saturated SKO for preservation of *A. bisporus* mushrooms to maintain tissue firmness, sensory attributes, reduce microorganism growth, slow down the degradation of phenolic compounds and ascorbic acid, ultimately extending the shelf life of the mushrooms. The findings demonstrated that TG coating with saturated SKO (TGSKO) helped retain 92.4% of tissue firmness and decreased the presence of microorganisms like molds, and *Pseudomonas*. Furthermore, the mushrooms coated with TGSKO exhibited a 57.1% decrease in the browning index, notably higher total phenolic content (85.6%) and ascorbic acid accumulation (71.8%) (15). Turmeric derived from a plant with medicinal applications is the most widely favored spice in culinary applications and is a prominent ingredient in various health products (170). Its biological properties include anti-inflammatory, anticancer, antioxidant, and antibacterial properties (171). After 15 days of storage, it was observed that mushrooms treated with turmeric essential oil exhibited increased firmness fewer changes in a browning and microbial population and displayed the most minimal activity of PPO, ascorbic acid and total phenols concentration (172). In short, biopolymer-based edible films, shaped through blending and water evaporation, offer an eco-friendly packaging solution for the rising demand for high-quality edible mushrooms. These films, infused with botanical extracts and essential oils (e.g., lemon, oregano, cumin etc.) enhance shelf life, preserve antioxidants, and inhibit microbial growth in mushrooms.

#### 5.3.7 Role of melatonin In mushroom preservation

**Figure 5:**
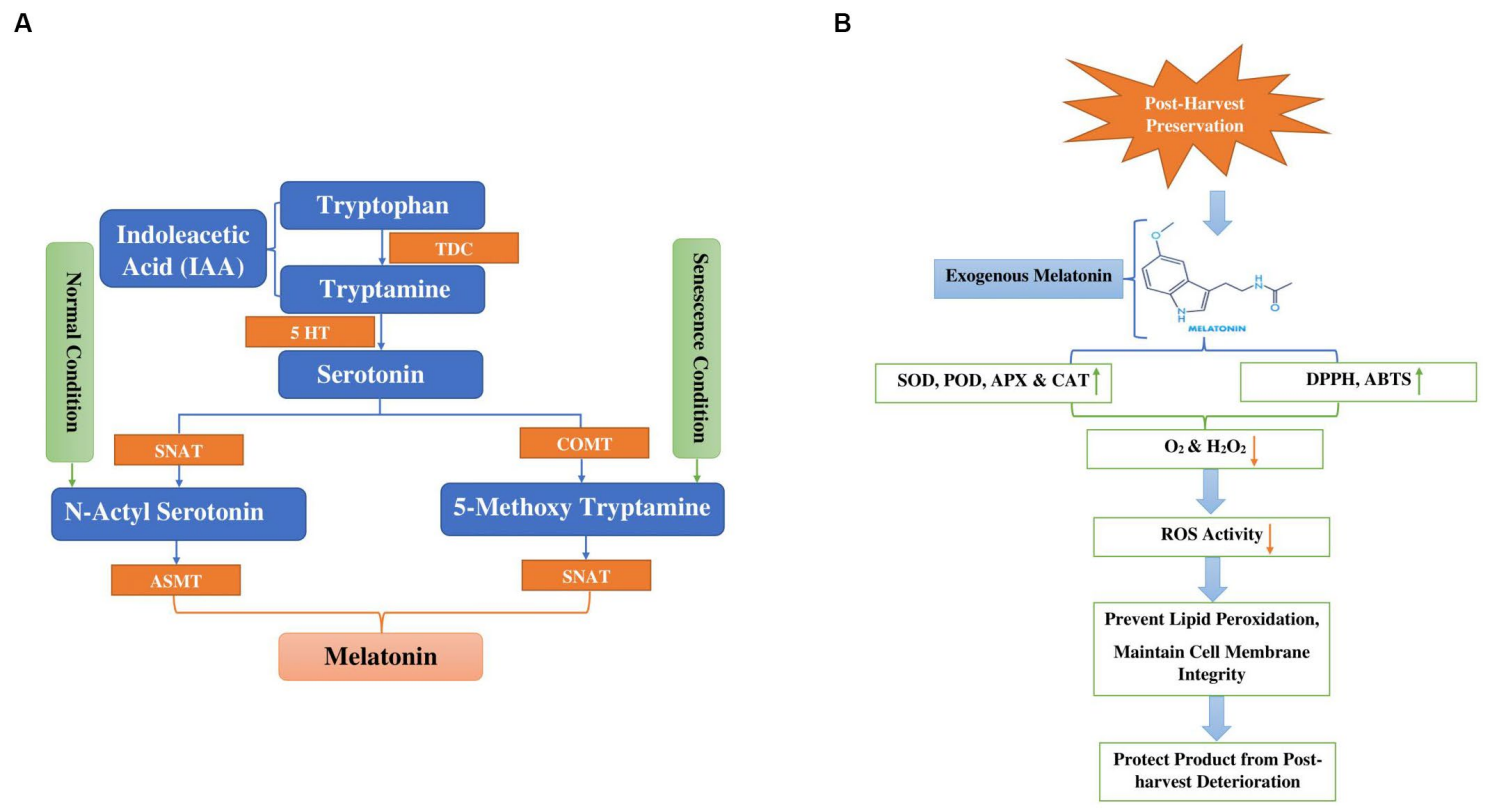
**(A)** Display melatonin biosynthesis in plants from tryptophan through hydroxylation and decarboxylation (TDC Tryptophan decarboxylase, 5HT 5Hydroxy L-tryptophan decarboxylase, COMT Caffeic acid O-methyltransferase, SNAT Serotonin N-acetyl transferase, ASMT N-acetyl serotonin methyltransferase). **(B)** Diagram depicts the mode of action of melatonin to alleviate chilling injury during cold storage and post-harvest deterioration green arrows indicated increase in antioxidant activity and orange arrows indicated decrease in ROS, O_2_ and H_2_O_2._ (DPPH 2,2-Diphenyl-1-picrylhydrazyl, ABTS 2,2′-azino-bis 3-ethylbenzothiazoline-6-sulfonic acid).

Melatonin (MT) is a multifaceted compound commonly found in nature (173). Its diverse functions impact not only animals and humans but also influence plant growth and development (174). Melatonin biosynthesis is enzymatically transformed, non-enzymatically or pseudo-enzymatically into numerous active metabolites such as AFMK (N1-acetyl-N2-formyl-5-methoxykynuramine), 5-MT (5-methoxytrayptamine), C3HMO (cyclic 3-hydroxymelatonin) and AMK N1-acetyl-5-methokynuramine (175). Melatonin, derived from the amino acid tryptophan, plays a widespread role in nature. Tryptophan functions not just as a precursor to melatonin but also as a substrate for indole-3-acetic acid (IAA), potentially contributing to the diverse functions observed in the plant system. Figure 5A illustrates pathways to melatonin synthesis. Two major melatonin pathways exist based on enzymes. One is the tryptophan/tryptamine/serotonin/N-acetylserotonin/melatonin pathway, which may occur under normal growth conditions. The other is the tryptophan/tryptamine/serotonin/5-methoxytryptamine/melatonin pathway, which may be activated when plants produce substantial amounts of serotonin, such as during senescence (176, 177). Firstly, tryptophan is transformed into two different products: tryptamine through the action of the tryptophan decarboxylase (TDC) enzyme, and 5-hydroxytryptophan via the participation of tryptophan hydroxylase (TPH) enzymes. Serotonin is transformed into N-acetyl-serotonin by the enzyme serotonin-5-acetyl transferase (SNAT). Subsequently, N-acetyl serotonin is ultimately converted into melatonin by the enzyme known as N-acylserotonin methyltransferase (ASMT) Tryptamine and 5-hydroxytryptophan are metabolized into serotonin through the activity of tryptamine 5-hydroxylase (T5H) and tryptophane decarboxylase (TDC) enzymes, respectively (175, 178, 179).

Freshly harvested vegetables constitute living material and they undergo various physiological and biochemical processes, including respiration, hormonal fluctuations, dehydration, and enzymatic changes (180). The application of exogenous melatonin prompted the endogenous melatonin biosynthetic process through antagonistic interaction with calcium, thereby safeguarding the product against chilling injury and postharvest deterioration, as indicated in Figure 5B. At room temperature storage, melatonin treatment reduced the relative membrane-leakage rate and inhibited the generation of superoxide radicals (O_2_), ethylene synthesis, hydrogen peroxide (H_2_O_2_) and malondialdehyde (MDA), resulting in prolonged freshness and a reduction in browning and degradation of chlorophyll/pigments in fruits and vegetables (176). The application of melatonin resulted in a decrease in the relative membrane-leakage rate and the inhibition of superoxide radicals (O_2_), malondialdehyde (MDA) and hydrogen peroxide (H_2_O_2_) production. Simultaneously, it increased the activities of antioxidant enzymes catalase (CAT), superoxide dismutase (SOD), glutathione reductase (GR) and ascorbate peroxidase (APX). Conversely, it decreased the activities of browning-related enzymes, such as polyphenoloxidase (PPO) and peroxidase (POD). The intervention also increased the expression of four genes responsible for encoding enzymes involved in the repair of oxidized proteins: LcMsrA1, LcMsrA2, LcMsrB1, and LcMsB2 (181). Li et al. (114) investigated the impact of exogenous melatonin at different concentrations (0.1, 0.2, and 0.05, mM) on the texture, sensory attributes, and electron leakage in white mushrooms stored at 3 ± 1°C. The study revealed that mushrooms treated with 0.1 mM melatonin exhibited superior quality, accompanied by a significant reduction in electron leakage. Moreover, the results indicated that 0.1 mM melatonin preserved higher adenosine triphosphate levels and prevented the release of cytochrome c into the cytoplasm. Notably, this concentration notably suppressed electron leakage by enhancing the activities of complexes I and III through the upregulation of AbRIP1 and AbNdufB9 genes. Conversely, Gao et al. (115) concentrated on improving mushrooms’ tolerance to cadmium by examining antioxidant-related metabolites and enzymes to confirm the beneficial impact of melatonin on Cd-induced oxidative stress. Their findings suggest that MT enhances the antioxidant activity of *Volvariella volvacea* through processes such as amino acid metabolism, glutathione metabolism, redox reactions, detoxification, and cellular oxidant detoxification, indicating that exogenous MT can shield edible mushrooms from Cd-induced oxidative stress. Hence, melatonin comprises various active components that selectively influence the autoxidation and antibacterial pathways during the preservation of edible mushrooms, rendering it a favorable option for their storage and preservation. Further investigation is needed to fully understand the effectiveness and best application methods of melatonin in improving the shelf life and quality of mushrooms.

## 6 Limitations and future prospects

Although the preservation techniques discussed above successfully retain certain quality traits of *Lentinula edodes* mushrooms, they have their limitations. For example, drying can extend shelf life but may change texture and color. Vacuum cooling maintains the heat-sensitive nature of fresh mushrooms without requiring substantial investment in vacuum systems. Irradiation methods require precise control of dosage rates within specified parameters. Chemical processes, such as washing mushrooms with antimicrobial agents or electrolyzed water, could temporarily elevate water activity, encouraging microbial growth.

Currently, the most effective approaches to preserving mushrooms include thermal processing, packaging, and chemical agents. Non-thermal methods such as ultrasound and cold plasma have had limited use in recent times. However, combining innovative techniques with conventional ones could enhance mushroom postharvest quality in the future. For example, employing cold plasma treatment alongside modified atmosphere packaging (MAP), or integrating ultrasound with cold plasma, offers potential for improving mushroom preservation. Delving into intricate molecular processes during preservation is vital for improving post-harvest quality, and melatonin treatment may be a pivotal solution on the horizon.

## 7 Conclusion

This review paper explores advancements in post-harvest preservation techniques for *Lentinula edodes* mushrooms. It focuses on quality degradation, factors influencing post-harvest quality, and preservation methods. Essential parameters like color, moisture content, texture, microbial counts, nutrients, and flavor can be maintained through suitable post-harvest preservation techniques. While physical, chemical, and thermal methods effectively prolong *Lentinula edodes* shelf life, internal quality deterioration remains inevitable. Encouraging the adoption of innovative techniques and combinations with low capital costs or shorter processing times is essential for further enhancing mushroom postharvest quality.

## Author contributions

HA: Conceptualization, Formal analysis, Resources, Writing – original draft, Writing – review & editing. FC: Formal analysis, Writing – review & editing. MH: Conceptualization, Writing – review & editing. AA: Conceptualization, Formal analysis, Validation, Writing – review & editing. SS: Formal analysis, Investigation, Writing – review & editing. MR: Formal analysis, Investigation, Project administration, Writing – review & editing. AT: Formal analysis, Methodology, Resources, Writing – review & editing. DW: Conceptualization, Supervision, Writing – original draft. YC: Supervision, Writing – original draft, Writing – review & editing. MAR: Writing - review & editing.
